# Systemic and Ocular Determinants of Choroidal Structures on Optical Coherence Tomography of Eyes with Diabetes and Diabetic Retinopathy

**DOI:** 10.1038/s41598-019-52750-0

**Published:** 2019-11-07

**Authors:** Takamasa Kinoshita, Hiroko Imaizumi, Miho Shimizu, Junya Mori, Akira Hatanaka, Shuichiro Aoki, Hirotomo Miyamoto, Masanori Iwasaki, Fumiko Murao, Masanori Niki, Hiroki Sano, Shozo Sonoda, Taiji Sakamoto, Yoshinori Mitamura

**Affiliations:** 1 Department of Ophthalmology, Sapporo City General Hospital, 1-1, North 11, West 13, Chuoku, Sapporo, 064-8604 Japan; 2 Department of Ophthalmology, Institute of Biomedical Sciences, Tokushima University Graduate School, 3-18-15 Kuramoto, Tokushima, 770-8503 Japan; 3 Department of Ophthalmology, Kagoshima University Graduate School of Medical and Dental Sciences, 8-35-1 Sakuragaoka, 890-8520 Kagoshima, Japan

**Keywords:** Retinal diseases, Diabetic nephropathy

## Abstract

Knowledgeof the choroidal structures in eyes with diabetes and diabetic retinopathy (DR) should provide information on the pathogenesis of DR. A prospective study was performed to determine the systemic and ocular factors that affect the choroidal structures in eyes with diabetes. Two-hundred consecutive diabetic subjects consisted of 160 treatment-naïve patients with different stages of DR and 40 patients with proliferative DR with prior panretinal photocoagulation (PRP). All underwent blood and urine tests and enhanced depth imaging optical coherence tomography (EDI-OCT). The cross-sectional EDI-OCT images of the subfoveal choroid were binarized to measure the total choroidal area (TCA), luminal area, and stromal area. Multivariate regression analyses were performed to determine the systemic and ocular factors that were significantly correlated with the choroidal structures. The subfoveal choroidal thickness, TCA, luminal area, and stromal area were larger at more advanced stage of DR, and smaller in eyes with PRP than those without (*P* < 0.001). The TCA and stromal area were significantly and positively correlated with the degree of albuminuria (*P* = 0.034, *P* = 0.025, respectively). The choroidal lumen and stroma may increase as the stages of DR progress and decrease after PRP. Albuminuria may be associated with the choroidal stromal edema.

## Introduction

Recent studies have shown that the choroid plays an important role in the physiology of the eye and the pathogenesis of different retinal diseases including diabetic retinopathy (DR)^1–5^. Because choriocapillaris supplies the oxygen and nutrients to the outer retinal layers, impairment of choriocapillaris can cause photoreceptor dysfunction and death.

The results of studies on choroidal thickness in eyes with diabetes and DR are still contradictory. Several studies reported there was a thinning of choroid as the disease progressed^1,2^, while others reported it thickened contrary^3^ or did not change^4,5^. One of the reasons for the discrepancies is that there are many potential confounders that can influence the choroidal thickness. These include age, refractive error, axial length, diurnal variations, prior ocular treatments including retinal photocoagulation, intravitreal injection of anti-vascular endothelial growth factors, intravitreal or subtenon injection of steroids, and intraocular surgeries^6–13^. In addition, duration of diabetes, status of glycemic control, severity of diabetic nephropathy, systemic hypertension, dyslipidemia, anemia, and medications to treat these conditions can also influence the choroidal vasculature and stroma^14–21^. Other limitations of previous studies include their retrospective nature and small sample size^1,4,5^.

There is good evidence for microvascular dropout and narrowing of the choroidal arterioles which are one of the characteristic features of the DR that can cause a thinning of choroid^22,23^. However, it may also be possible that increased vascular permeability, another characteristic feature of the DR, can cause a swelling of the choroidal stroma and thickening of the choroid^3^. Thus, it would be useful to evaluate the vascular and stromal components of the choroid separately to understand the pathophysiology of DR.

A method using the binarization of enhanced depth imaging optical coherence tomographic (EDI-OCT) images or swept source OCT images can differentiate and quantify the choroidal luminal area and the stromal area with high repeatability and reproducibility in normal and diseased eyes^8,9,24,25^.

Thus, the purpose of this study was to prospectively evaluate the choroidal structures in patients with diabetes with and without DR using binarization of EDI-OCT images and to determine the systemic and ocular factors that were significantly correlated with the choroidal structures in consideration of extensive potential confounders..

## Results

Two hundred and forty consecutive potentially eligible Japanese subjects were invited to participate in the study until the number of cases in each group reached 40. Two subjects declined to participate in the study. Of 238 subjects who agreed to participate in the study, 38 patients were excluded because of the exclusion criteria; 15 subjects with high myopia, 10 with poor OCT image quality, 5 with macular photocoagulation, 4 with prior vitrectomy, 2 with pregnancy, and 2 with severe anemia. In the end, 200 eyes of 200 subjects consisting of 126 men and 74 women with a mean (standard deviation) age of 58.6 (12.85) years were studied.

### Baseline demographic data

Baseline demographic data are summarized in Supplementary Table S1. There were significant differences in several demographic data including the age among the different stages of DR. In the group of proliferative DR (PDR) with prior panretinal photocoagulation (PRP), PRPs were performed more than 6 months before enrollment in all eyes.

### Correlations of choroidal parameters with age, axial length, and sex

The subfoveal choroidal thickness (SFCT), total choroidal area (TCA), luminal area, and stromal area were significantly and negatively correlated with age and axial length (Supplementary Table S2). The ratio of luminal area to stromal area (L/S ratio) was negatively correlated with the axial length. The SFCT, TCA, luminal area, and stromal area were not significantly different between sexes without any adjustments, but they were significantly larger in men than in women after adjustments for the age and axial length (Supplementary Table S3).

### Differences in the choroidal structures at different stages of diabetic retinopathy

**Figure 1:**
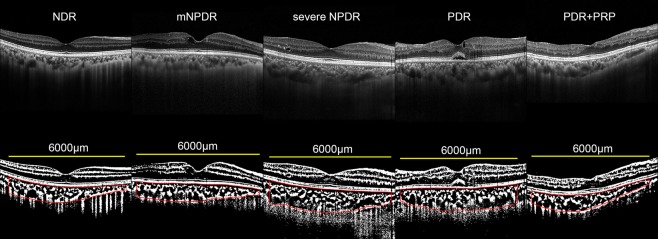
Enhanced depth imaging optical coherence tomographic (EDI-OCT) images and binarized images of EDI-OCT acquired from eyes with different stages of diabetic retinopathy. (Top row). EDI-OCT images. (Bottom row) binarized EDI-OCT images. Subfoveal choroidal thickness and total choroidal area were significantly larger at more advanced stage of DR, and smaller in eyes with PRP than those without. The margins of the region of interest are delineated by red lines. Yellow bars indicate the length of 6000 μm. NDR, no diabetic retinopathy; mNPDR, mild to moderate nonproliferative diabetic retinopathy; sNPDR, severe NPDR; PDR, proliferative diabetic retinopathy; PRP, panretinal photocoagulation.

The SFCT, luminal area, and stromal area were significantly larger in eyes with severe non-proliferative DR (sNPDR) and PDR without PRP than those with no diabetic retinopathy (NDR) with and without correction for age and axial length (Fig. 1, Table 1, Supplementary Fig. 1). The SFCT, TCA, luminal area, and stromal area in the eyes with PDR with prior PRP were significantly smaller than those in the eyes with PDR without PRP with and without correction for age and axial length. There was no significant difference in the L/S ratio among the eyes at different stages of DR.

**Table 1:** Differences in the choroidal structures among the eyes with different stages of diabetic retinopathy.

	NDR	mNPDR	severe NPDR	PDR without PRP	PDR with PRP	*P* value
SFCT, mean (SEM) [95% CI], μm, unadjusted	255.0(10.63) [233.5–276.5]	283.0 (12.06) [258.6–307.4]	303.4 (13.56) [275.9–330.8]	345.9 (12.95) [319.7–372.1]	253.1 (11.35) [230.2–276.1]	<0.001*
adjusted	253.5 (10.55) [232.7–274.3]	294.1 (10.70) [273.0–315.3]	308.9 (10.55) [288.1–329.7]	330.3 (10.75) [309.1–351.5]	253.5 (10.52) [232.8–274.3]	<0.001^†^
Total choroidal area, mean (SEM) [95% CI], 10^3^ μm^2^, unadjusted	1484.4 (57.87) [1367.3–1601.4]	1613.5 (64.39) [1483.3–1743.8]	1717.3 (68.75) [1578.3–1856.4]	1956.6 (70.14) [1814.7–2098.4]	1456.3 (60.29) [1334.3–1578.2]	<0.001*
adjusted	1472.9 (56.08) [1362.2–1583.5]	1679.2 (56.88) [1567.0–1791.4]	1747.7 (57.15) [1637.1–1858.3]	1869.2 (57.15) [1756.5–1981.9]	1459.1 (55.95) [1348.7–1569.4]	<0.001^†^
Luminal area, mean (SEM) [95% CI], mean (SD), 10^3^ μm^2^, unadjusted	981.7 (41.46) [899.9–1063.4]	1134.9 (42.05) [1052.0–1217.8]	1150.7 (41.46) [1068.9–1232.4]	1235.6 (42.25) [1152.3–1318.9]	961.0 (41.36) [879.4–1042.6]	<0.001*
adjusted	974.3 (42.57) [890.3–1058.3]	1121.3 (43.20) [1036.1–1206.5]	1154.8 (42.71) [1070.6–1239.0]	1195.7 (43.55) [1109.8–1281.6]	997.9 (42.43) [914.2–1081.6]	<0.001^†^
Stromal area, mean (SEM) [95% CI], 10^3^ μm^2^, unadjusted	491.2 (16.64) [458.4–524.1]	544.3 (16.88) [511.0–577.6]	597.0 (16.64) [564.2–629.9]	633.6 (16.96) [600.2–677.1]	498.1 (16.60) [465.4–530.9]	<0.001*
adjusted	488.2 (16.41) [455.8–520.5]	539.0 (16.65) [506.1–571.8]	580.4 (16.46) [548.0–612.9]	621.2 (16.79) [588.1–654.3]	496.9 (16.36) [464.6–529.2]	<0.001^†^
Luminal/Stromal ratio (SEM) [95% CI], unadjusted	1.982 (0.035) [1.911–2.052]	2.073 (0.039) [1.994–2.153]	1.930 (0.041) [1.847–2.013]	1.965 (0.044) [1.876–2.054]	1.917 (0.044) [1.829–2.005]	0.061*
adjusted	1.986 (0.040) [1.907–2.065]	2.072 (0.040) [1.993–2.150]	1.932 (0.040) [1.854–2.011]	1.943 (0.040) [1.882–2.039]	1.916 (0.040) [1.837–1.995]	0.057^‡^

NDR, no diabetic retinopathy; mNPDR, mild to moderate nonproliferative diabetic retinopathy; PDR, proliferative diabetic retinopathy; PRP, panretinal photocoagulation; SFCT, subfoveal choroidal thickness; SEM, standard error of the mean.

*One-way analysis of variance.

^†^General linear model where age and axial length were used as covariance. Covariates appearing in the model are evaluated at the following values: age = 58.58, axial length = 23.86.

^‡^General linear model where axial length were used as covariance. Covariates appearing in the model are evaluated at the following values: axial length = 23.86.

### Correlations between choroidal parameters and continuous variables

The SFCT, TCA, luminal area, and stromal area were significantly and positively correlated with the albumin index in the partial regression coefficient (Supplementary Table S2). The stromal area was also significantly correlated with the serum level of albumin. The L/S ratio was significantly correlated with the best-corrected visual acuity (BCVA) and central macular thickness (CMT).

### Differences in the choroidal structures among groups of different categorical variables

Patients were classified as having systemic hypertension when their blood pressure was > 140/90 mmHg, or were being treated with anti-hypertensive medications. The TCA and luminal area were significantly smaller in the eyes with systemic hypertension than normotensive subjects (Supplementary Table S4). The SFCT, TCA, and stromal area in eyes with diabetic macular edema (DME) were larger than those without DME. There were no significant differences in the choroidal parameters between using or not using the different types of medications for hypertension, hyperglycemia, and dyslipidemia (Supplementary Tables S5 and S6).

### Evaluating factors influencing choroidal parameters in multivariate regression analyses

Using the variables which were significant in univariate analysis for each choroidal parameter, multivariate regression analyses were performed. In multivariate regression analyses, the SFCT was significantly associated with the age, axial length, sex, and the stage of DR. The TCA was significantly associated with the age, axial length, sex, albumin index, the stage of DR, and hypertension. The luminal area was significantly associated with the age, axial length, sex, the stage of DR, and hypertension. The stromal area was significantly associated with the age, axial length, albumin index, and the stage of DR. The L/S ratio was significantly correlated with the axial length and BCVA. (Table 2).

**Table 2:** Multivariate Regression Analysis for the determinants that influenced the choroidal structures*.

	SFCT, B (β) [95% CI], μm	p	Total choroidal area, B (β) [95% CI], 10^3^ μm^2^	p	Luminal area, B (β) (95% CI), 10^3^ μm^2^	p	Stromal area, B (β) (95% CI), 10^3^ μm^2^	p	L/S ratio, B (β) (95% CI)	p
Adjusted R^2^	0.390		0.375		0.338		0.413		0.084	
Age	−2.7 (−0.417) [−3.5 to −1.9]	<**0.001**	−14.6 (−0.423) [−18.8 to −10.4]	<**0.001**	−9.8 (−0.396) [−12.8 to −6.7]	<**0.001**	−4.9 (−0.455) [−6.1 to −3.6]	<**0.001**		
axial length	−34.8 (−0.414) [−44.9 to −24.8]	<**0.001**	−165.6 (−0.372) [−218.3 to −112.9]	<**0.001**	−121.6 (−0.382) [−160.3 to −82.8]	<**0.001**	−45.2 (−0.326) [−61.7 to −28.6]	<**0.001**	−0.054 (−0.206) [−0.089 to −0.019]	**0.003**
Sex (women)	−23.4 (−0.135) [−43.9 to −2.9]	**0.026**	−145.3 (−0.159) [−253.2 to −37.3]	**0.020**	−112.3 (−0.172) [−191.7 to −32.9]	**0.006**	−27.9 (−0.098) [−61.5 to 5.7]	0.103		
Albumin index†	0.004 (0.087) [−0.002 to 0.010]	0.160	34.3 (0.130) [2.6 to 66.0]	**0.034**	22.1 (0.117) [−1.2 to 45.3]	0.063	14.5 (0.177) [1.8 to 27.2]	**0.025**		
Stage of DR, NDR	0		0		0		0			
mNDR	35.9 (0.173) [6.8 to 65.1]	**0.016**	200.2 (0.181) [46.3 to 354.1]	**0.011**	164.7 (0.209) [52.0 to 277.3]	**0.004**	30.6 (0.089) [−17.0 to 78.1]	0.206		
Severe NPDR	53.9 (0.239) [22.6 to 84.8]	**0.001**	284.9 (0.240) [120.2 to 449.7]	<**0.001**	204.4 (0.240) [85.5 to 323.3]	**0.001**	81.4 (0.220) [30.7 to 132.2]	**0.002**		
PDR without PRP	54.2 (0.258) [21.9 to 86.5]	**0.001**	284.8 (0.256) [114.0 to 455.7]	**0.002**	195.3 (0.245) [75.6 to 315.0]	**0.002**	89.9 (0.259) [37.0 to 142.7]	**0.001**		
PDR with PRP	−4.7 (−0.023) [−34.0to 24.6]	0.753	−4.7 (−0.004) [−161.8 to 152.4]	0.758	10.7 (0.014) [−103.6 to 125.1]	0.853	−28.3 (−0.083) [−76.7 to 20.1]	0.250		
DME (present)	4.5 (0.026) [−16.7 to 25.8]	0.673	33.4 (0.036) [−78.0 to 144.9]	0.555			−45.4 (−0.158) [−130.0 to 39.5]	0.292		
Hypertension (present)			−132.5 (−0.139) [−246.3 to −18.6]	**0.023**	−98.9 (−0.145) [−182.5 to −15.4]	**0.021**				
Type of DME, absent							0			
Sponge-like swelling							9.1 (0.021) [−58.8 to 77.1]	0.791		
Cystoid macular edema							74.5 (0.224) [−14.0 to 163.0]	0.098		
Serous retinal detachment							59.4 (0.116) [−34.8 to 153.6]	0.215		
Serum albumin							12.2 (0.051) [−24.9 to 49.4]	0.516		
BCVA									−0.198 (−0.174) [−0.363 to 0.032]	**0.019**
CMT									−0.000 (−0.108) [−0.001 to 0.000]	0.144

Using the variables which were significant in univariate analysis for each choroidal parameter, multivariate regression analyses were performed. Values in bold indicate statistical significance (*P* < 0.05). SFCT, subfoveal choroidal thickness; L/S ratio, ratio of luminal to stromal area; eGFR, estimated glomerular filtration rate; NDR, no diabetic retinopathy; mNPDR, mild to moderate nonproliferative diabetic retinopathy; PDR, proliferative diabetic retinopathy; PRP, panretinal photocoagulation; BCVA, best corrected visual acuity; CMT, central macular thickness.

*Data were presented as B, unstandardized coefficients (β, standardized coefficients) [95% CI for B].

^†^Albumin index = the ratio of urinal albumin to urinal creatinine.

## Discussion

The results showed that the SFCT, TCA, luminal area, and stromal area were significantly associated with the age, axial length, and sex in the eyes of diabetic patients with or without DR. These finding are in agreement with earlier reports in normal eyes^6–8^. Importantly, the age and axial length had the strongest correlation with the size of the choroidal structures in the multivariate regression analyses. Thus, the choroidal structures in eyes with diabetes and DR should be evaluated under strict control of the age and axial length. Unfortunately, as best we know, only a few studies have evaluated the choroidal structures in such a manner which may be one of the reasons for the contradictory results of the choroidal thickness in DR.

Histopathological studies have shown that a dropout of the choriocapillaris was more extensive in diabetic than that in non-diabetic eyes^22^. Other studies showed that the blood flow of the choriocapillaris was reduced in eyes with diabetes and DR^26–28^, and the degree of reduction increased as the severity of DR progressed^27,28^. As the disease progresses, the increased dropout of choriocapillaris and subsequent hypoxia of retinal pigment epithelium (RPE) and photoreceptors increase the expression of vascular endothelial growth factor (VEGF) from the RPE cells^29,30^ which would lead to a vasodilation of middle and large choroidal vessels. In experimental animals, VEGF increased the vessel diameter^31^, and in human, anti-VEGF therapy decreased the choroidal luminal area in eyes with DME and polypoidal choroidal vasculopathy^25,32^. Pulsatile ocular blood flow which reflects the whole choroidal blood flow was reported to increase as the severity of retinopathy progressed^33,34^. These findings may explain why the luminal area was larger in sNPDR and PDR without PRP than in NDR.

The increase in the vascular permeability caused by increased levels of VEGF induces vascular leakage and swelling of the choroidal stroma. Choroidal vascular hyperpermeability and leakage of proteinaceous fluid into the choroidal stroma have been reported in the eyes with DR^3,23^. These changes are compatible with our results that the stromal areas in sNPDR and PDR without PRP were larger than that in NDR. Thus, the increase of both the luminal and stromal areas with a progression of DR account for the increase in the SFCT and TCA without significant changes in the L/S ratio.

The SFCT, TCA, luminal area, and stromal area in eyes with PDR with PRP were smaller than those in the eyes with PDR without PRP. Previous studies reported that the choroidal thickness, total choroidal area, luminal area as well as the choroidal blood flow were decreased after the PRP^10,25,33^. It has been reported that the VEGF level in ocular fluid in patients with PDR was higher than that in patients with NPDR, and that it was decreased after PRP^35^. These findings suggest that the PRP reduced the expression of VEGF which then led to a reduction of the choroidal vessel diameters and vascular permeability resulting in a thinning of the choroid. However, the effects of PRP on the choroidal structures remain to be elucidated because other cytokines and inflammatory cells are known to be involved in this complicated condition^36^.

We found no significant association between choroidal parameters and the presence of DME or type of DME in multivariate analysis. In several studies that examined the choroidal thickness in eyes with DME and/or PDR, subjects included had undergone prior treatment with PRP or anti-VEGF therapy that reduces the choroidal thickness. This may be another reason for the contradictory results of the choroidal thickness in DR.

The albumin index was significantly correlated with the choroidal stromal area. The reason for the results remains unclear. A possible explanation would be that hypoalbuminemia caused by albuminuria lead to fluid accumulation in the stromal tissue in the body including choroidal stroma; however, choroidal stromal area was not significantly associated with serum albumin in the multivariate analysis.

Albuminuria is caused by the disruption of the filter barrier in the glomeruli which are comprised of negatively charged barriers of the endothelial cells of the glomerular capillaris and basement membranes, and the size barriers of the podocytes^37,38^. The negatively charged barriers restrict the passage of negatively charged molecules such as albumin through the capillary lumens into Bowman’s capsule. Choriocapillaris which has fenestration similar to the glomerular capillaries consists of endothelial cells lined with a basement membrane both of which are negatively charged and serve as charge barriers^39,40^. It has been reported that there is a disruption of filtration barrier associated with a thickening of the basement membrane and a decrease in anionic sites in the basement membrane of the choriocapillaris and the glomeruli in diabetic patients^41,42^. Histologically, the choroid in diabetic choroidopathy and the glomeruli in diabetic nephropathy have the characteristic features in common including the thickening of the basement membrane of capillaries associated with the periodic acid–Schiff (PAS)-positive materials^23,43^. PAS-positive nodules in the choroidal stroma were reported to closely resemble those in the mesengial matrix of glomeruli (Kimmelstiel-Wilson nodule)^23^. It might be possible that when the glomerular filter barrier is damaged more severely, there is a more extensive breakdown of the charge barrier of choriocapillaris which has structural and functional similarity to the glomeruli. The breakdown of the charge barrier of the choriocapillaris would allow large molecules to leak outside the capillaries, resulting in fluid retention in the choroidal stroma. This may explain the association between the albuminuria and the choroidal stromal edema.

The TCA and luminal area were significantly smaller in the patients with systemic hypertension than normotensive subjects. Choroidal blood volume measured with laser Doppler Flowmetry has been reported to be reduced in patients with systemic hypertension^16^. In addition, choroidal thickness has also been reported to be reduced in patients with systemic hypertension or coronary artery disease^17,44^. The decrease in the luminal area caused by arteriosclerosis or vascular contraction due to systemic hypertension may lead to the decrease in the TCA. In contrast, Ahn *et al*. reported that the choroidal thickness was increased in patients with severe hypertension (defined as systolic blood pressure > 180 or diastolic blood pressure > 110)^18^. They assumed that interstitial fluid accumulation caused by the increased choroidal permeability due to extremely high blood pressure led to the thickening of the choroid. In our study, only 12 (8.7%) of 138 patients with systemic hypertension had such extremely high blood pressure. Thus, we considered that the effect of increased choroidal permeability on the choroidal thickness or choroidal stroma was not observed.

The L/S ratio was negatively correlated with axial length, which was consistent with previous reports^8^. It also was negatively correlated with BCVA. The reason for the results was not unclear; however, the impact of these factors on the L/S ratio was quite small (adjusted R^2^ = 0.084). Other factors which were not evaluated in this study might be associated with the L/S ratio.

There are limitations in this study. First, the evaluations of the cross-sectional images of 6000 μm were probably not sufficient to assume the structural changes of the entire choroid. Evaluations of three-dimensional volumetric scans may provide more conclusive data. Second, this was a cross-sectional study and was limited in evaluating the influence of some variants on the choroid especially the effects of medications for systemic hypertension and hyperglycemia on the choroidal structures. Longitudinal studies are needed in the future. Third, the manual delineation of the chorioscleral interface was not completely objective. Lastly, the choroidal structural changes observed on the OCT images should be interpreted carefully, because we did not confirm them histologically. The strength of this study included its prospective design in consideration of extensive potential confounders in treatment-naïve eyes.

In conclusion, the subfoveal choroidal thickness, total choroidal area, luminal area, and stromal area in eyes with diabetes may increase with the progression of DR but decrease after PRP. The total choroidal area and stromal area were significantly correlated with the degree of albuminuria, suggesting the association between the albuminuria and choroidal stromal edema.

## Material and Methods

The procedures used in this study conformed to the tenets of the Declaration of Helsinki, and a signed informed consent was obtained from each subject. This study was approved by the Institutional Review Boards of Sapporo City General Hospital and Tokushima University Hospital.

### Inclusion and exclusion criteria

This was a prospective, cross-sectional, interventional study of 200 eyes of 200 consecutive patients with type 1 or type 2 diabetes who were examined at Sapporo City General Hospital and Tokushima University Hospital between February 2017 and January 2019. During the period, consecutive potentially eligible Japanese outpatients with diabetes were invited to participate in the study until the number of cases in each group reached 40. The right eye of each patient was included. When the right eyes were excluded by the exclusion criteria, the left eyes could be included.

The exclusion criteria included the age of < 20 years or > 80 years, high myopia defined as a spherical equivalent of < −6.0 diopters or an axial length of > 26.5 mm, a BCVA worse than 20/400, ocular hypertension defined as intraocular pressure of > 24 mmHg, corneal diseases, glaucoma, and chorioretinal diseases other than DR. Eyes with cataract, premacular hemorrhage, and severe macular hard exudates that affected the BCVA and quality of EDI-OCT images, poor EDI-OCT image quality defined as an image quality index of < 30, abnormal EDI-OCT findings from other chorioretinal diseases than DR were also excluded. Eyes that had been treated with intravitreal injections of anti-VEGF, intravitreal or subtenon injection of steroids, macular photocoagulation, and previous intraocular surgeries except for non-complicated cataract surgery also were excluded. Eyes treated with any type of previous retinal photocoagulation were excluded except for the eyes with PDR with PRP which were performed more than 6 months before enrollment. Patients with other systemic diseases such as heart failure, malignancy, endocrine diseases, and severe anemia defined as hemoglobin < 8 g/dl, and subjects under hemodialysis, pregnancy, or steroidal treatment also were excluded.

### Systemic and ophthalmic examinations

Systemic examinations performed included systolic and diastolic blood pressure, mean arterial blood pressure (MAP), mean ocular perfusion pressure (MOPP), heart rate, hemoglobin, hemoglobin A1_C_, body mass index, serum level of albumin, triglycerides, total cholesterol, low density lipoprotein cholesterol, high density lipoprotein cholesterol, creatinine, and estimated glomerular filtration rate. MAP and MOPP were calculated according to the following formulas: MAP = diastolic blood pressure + 1/3 (systolic blood pressure – diastolic blood pressure), and MOPP = 2/3MAP- intraocular pressure. The degree of albuminuria was defined as the ratio of urinal albumin to urinal creatinine which was called albumin index. Information on the duration of diabetes and current smoking were collected from patients’ oral reports, and current medications for systemic hypertension, hyperglycemia, and dyslipidemia also were recorded according to the physician’s letter or the medical diaries that were generally used in Japan to record the all medications for each patient.

Ophthalmic examinations included measurements of the refractive error, axial length, the BCVA, and intraocular pressure, slit-lamp biomicroscopy, indirect ophthalmoscopy, fundus photography, and EDI-OCT. Fluorescein angiography was performed according to the ophthalmologists’ decision.

The stage of DR was classified according to the international classification with slight modifications^45^. We classified DR into following 5 groups; NDR, mild to moderate non-proliferative DR, sNPDR, PDR without prior photocoagulation, and PDR with prior PRP. DME was defined as increased retinal thickness due to DR with CMT ≥ 320 μm for men or ≥ 305 μm for women in a 1 mm diameter circle centered on the fovea, and was classified into 3 groups; sponge-like retinal swelling, cystoid macular edema, and serous retinal detachment as reported (Supplementary Table S1)^46^. Eyes with retinal swelling caused by epiretinal membrane or vitreomacular traction were excluded.

### Enhanced depth imaging optical coherence tomography

Horizontal and vertical cross-sectional EDI-OCT images of 30 degrees that passed through the fovea were obtained by Spectralis OCT instrument (Heidelberg Engineering, Heidelberg, Germany) for each eye. Each image was recorded through dilated pupil with the eye tracking system, and 100 scans were averaged to enhance the signal-to-noise ratio. The CMT was defined as the mean distance between the internal limiting membrane and the outer surface of the RPE in Early Treatment of Diabetic Retinopathy Study central subfield of 1mm-diameter. It was automatically measured following a raster scan composed of 31 horizontal B scans covering a 30 × 25 degrees area centered on the fovea.

The examined area of the choroid was 6000 μm wide and was centered on the fovea. The parameters measured were the SFCT, TCA, luminal area, and stromal area. The L/S ratio was calculated. The SFCT was defined as the distance between the outer border of the RPE and the chorioscleral interface at the center of the fovea, and it was measured by two independent investigators (TK and JM) using the caliper function of the software embedded in the Spectralis. The average of measurements on the horizontal and vertical scan images was defined as SFCT measured by each investigator, and the average of the measurements by two investigators was used for statistical analyses.

### Evaluation of choroidal areas by binarization technique

The EDI-OCT images were evaluated by one of the authors (JM) who was masked to the clinical findings. The binarization of the choroidal area in the EDI-OCT images was done by a modified Niblack method using the ImageJ software (ImageJ version 1.47, NIH, Bethesda, MD, USA) as described in detail (Fig. 1)^24^. The examined area was 6000 μm wide and centered on the subfoveal choroid from the RPE to the chorioscleral border. In the binarized images, the light and dark pixels were defined as the stromal and luminal areas. They were automatically calculated after adding the data on the relationship between the distance on the fundus and the pitch of the pixels in the EDI-OCT images, which was dependent on the axial length.

All parameters of each horizontal and vertical image were measured three times, and the averages of 6 measurements were used for the statistical analyses. The correlations of the choroidal parameters of EDI-OCT with ocular and systemic parameters were determined.

### Statistical analyses

The sample size calculation was performed with a statistical software G*Power 3.9.9.2 (Germany). The effect size of stage of DR on the choroidal thickness was set as 0.25 (medium size effect) according to the report by Cohen^47^ and previous contradictory results^1–5^. Using a two-sided alpha error of 5% and a power of 80%, 40 eyes were required for each of the 5 groups. Other statistical analyses were performed with the SPSS version 22 software (IBM, Armonk, New York, USA). The equalities of variances were confirmed with the Leven’s tests. The BCVA were converted from decimal visual acuity to the logarithm of the minimum angle of resolution for statistical analysis. The significance of the differences in the choroidal parameters between 2 groups was determined using independent *t*-tests, and the significances of differences among 3 or more groups were determined using one-way analysis of variance and general linear model with the Bonferroni test for post hoc analyses. The correlations between choroidal parameters and other continuous variables were determined by partial regression coefficients of correlation. Multivariate regression analyses were performed to determine the parameters that were significantly correlated with choroidal parameters. The time of day when the EDI-OCT images were obtained was evaluated by Kruskal-Wallis analysis^9^. A two-sided *P* value of < 0.05 was considered statistically significant

## Supplementary information

Supplementary file

## Data Availability

The dataset generated and analyzed in this study are available from the corresponding author upon reasonable request.
