# Supplementary material for: Systemic and Ocular Determinants of Choroidal Structures on Optical Coherence Tomography of Eyes with Diabetes and Diabetic Retinopathy
Source: Sci Rep. 2019 Nov 7;9:16228. doi: 10.1038/s41598-019-52750-0 (PMC6838057; doi:10.1038/s41598-019-52750-0)
Supplement: Supplementary file 1 — Supplementary file [file 41598_2019_52750_MOESM1_ESM.pdf]

Systemic and Ocular Determinants of Choroidal Structures on Optical Coherence Tomography of Eyes with Diabetes and Diabetic Retinopathy

Takamasa Kinoshita[1,2], Hiroko Imaizumi[1,2], Miho Shimizu[1], Junya Mori[1], Akira Hatanaka[1], Shuichiro Aoki[1], Hirotomo Miyamoto[1], Masanori Iwasaki[1], Fumiko Murao[2], Masanori Niki[2], Hiroki Sano[2], Shozo Sonoda[3], Taiji Sakamoto [3], Yoshinori Mitamura[2]

[1]Department of Ophthalmology, Sapporo City General Hospital, Sapporo, Japan.

[2]Department of Ophthalmology, Institute of Biomedical Sciences, Tokushima University Graduate School, Tokushima, Japan

[3]Department of Ophthalmology, Kagoshima University Graduate School of Medical and Dental Sciences, Kagoshima, Japan

Correspondence: Takamasa Kinoshita, Department of Ophthalmology, Sapporo City General Hospital, 1-1, North 11, West 13, Chuoku, Sapporo, 064-8604, Japan.

Tel: +81 11 726 2211; Fax: +81 11 726 9541

E-mail: knst129@gmail.com

Supplementary Table S1. Demographic information of 200 study participants

	All participants (n=200)	NDR (n=40)	mNPDR (n=40)	severe NPDR (n=40)	PDR without PRP (n=40)	PDR with PRP (n=40)	*P* value
Age, mean (SD) , y	58.6 (12.85)	56.9 (12.78)	63.6 (11.33)	60.3 (12.71)	53.2 (12.89)	59.0 (12.66)	0.004*
Sex, No. (%) women	74 (37.0)	16 (40.0)	16 (40.0)	15 (37.5)	11 (27.5)	16 (45.0)	0.719†
Type of diabetes, No. (%), type 1	15 (7.5)	6 (15.0)	1 (2.5)	2 (5.0)	1 (2.5)	5 (12.5)	0.116†
BCVA, mean (SD), logMAR	0.08 (0.229)	-0.69 (0.066)	0.02 (0.140)	0.13 (0.253)	0.16 (0.234)	0.17 (0.285)	<0.001‡
Axial length, mean (SD), mm	23.9 (0.99)	23.8 (0.71)	23.8 (1.02)	23.9 (1.02)	23.8 (1.04)	23.8 (1.16)	0.945*
Central macular thickness, mean (SD), µm	311.3 (87.40)	273.8 (20.33)	287.0 (41.27)	331.9 (106.60)	357.8 (112.39)	305.8 (90.19)	<0.001‡
Type of DME** absent	130 (65.0)	40 (100)	29 (72.5)	21 (52.5)	14 (35.0)	26 (65.0)	<0.001†
Sponge-like swelling	9 (4.5)	0 (0)	1 (2.5)	3 (7.5)	5 (12.5)	0 (0)	
Cystoid macular edema	42 (21.0)	0 (0)	9 (22.5)	9 (22.0)	11 (27.5)	13 (32.5)	
Serous retinal detachment	19 (9.5)	0 (0)	1 (2.5)	7 (17.5)	10 (25.0)	1 (2.5)	
Hypertension, No. (%), present	138 (69.0)	19 (47.5)	28 (70.0)	28 (70.0)	29 (72.5)	34 (85.0)	0.009†
Hemoglobin A1c, mean (SD), %	8.3 (2.10)	8.5 (2.33)	8.4 (2.12)	8.2 (2.39)	8.6 (2.17)	7.7 (1.24)	0.367*
Duration of	14.4 (12.08)	6.5 (6.93)	17.0 (9.33)	13.9 (9.76)	11.9 (13.79)	22.7 (13.32)	<0.001‡

diabetes, mean (SD), y							
Serum albumin, mean (SD), g/dL	4.0 (0.57)	4.2 (0.45)	4.1 (0.44)	4.1 (0.57)	3.8 (0.68)	4.0 (0.61)	0.013*
Serum creatinine (SD), mg/dl	0.75 (0.37)	1.02 (1.11)	0.89 (0.43)	1.32 (1.51)	1.31 (0.97)	1.06 (0.99)	0.001‡
eGFR, mean (SD), ml/min/1.73m^2	71.6 (31.86)	85.5 (28.42)	71.1 (29.56)	76.1 (30.53)	67.2 (35.1)	58.1 (30.25)	0.002*
Albumin index††, mean (SD), mg/gCr	811.5 (1679.39)	167.3 (481.71)	394.3 (1174.50)	478.1 (1140.67)	1842.2 (2534.5)	1175.5 (1763.8)	<0.001‡
Time of the day when OCT was performed, median (SD), time	11:24 (1:57)	11:03 (1:50)	11:53 (2:08)	11:06 (1:51)	11:04(1:49)	11:52 (2:00)	0.072‡

NDR, no diabetic retinopathy; mNPDR, mild to moderate nonproliferative diabetic retinopathy; PDR, proliferative diabetic retinopathy; PRP, panretinal photocoagulation; BCVA, best corrected visual acuity; logMAR, logarhithm of the minimum angle of resolution; eGFR, estimated glomerular filtration rate; DME, diabetic macular edema; OCT, optical coherence tomography. *One-way analysis of variance †Fisher's exact test ‡Kruskal-Wallis analysis

**Eyes with concurrent serous retinal detachment and other type of macular edema were categorized into serous retinal detachment group, and eyes with sponge-like swelling and cystoid macular edema were categorized into cystoid macular edema group.

†† Albumin index = the ratio of urinal albumin to urinal creatinine

Supplementary Table S2. Correlations of choroidal parameters with different continuous variables.

	SFCT	Total choroidal area	Luminal area	Stromal area	L/S ratio
Age	r = -0.412<br>p <0.001*	r = -0.441<br>p <0.001*	r = -0.404<br>p <0.001*	r = -0.485<br>p <0.001*	r = -0.002<br>p = 0.979*
Axial length	r = -0.391<br>p <0.001†	r = -0.353<br>p <0.001†	r = -0.351<br>p <0.001†	r = -0.323<br>p <0.001†	r = -0.202<br>p <0.001†
Intraocular pressure	r = -0.089<br>p = 0.215‡	r = -0.091<br>p = 0.204‡	r = -0.086<br>p = 0.228‡	r = -0.092<br>p = 0.197‡	r = -0.009<br>p = 0.902*
BCVA	r = 0.049<br>p = 0.497‡	r = 0.033<br>p = 0.647‡	r = -0.010<br>p = 0.885‡	r = 0.132<br>p = 0.065‡	r = -0.220<br>p = 0.002*
Central macular thickness	r = 0.017<br>p = 0.813‡	r = 0.016<br>p = 0.824‡	r = -0.020<br>p = 0.784‡	r = 0.099<br>p = 0.167‡	r = -0.179<br>p = 0.011*
Systolic blood pressure	r = 0.005<br>p = 0.939‡	r = 0.014<br>p = 0.844‡	r = 0.006<br>p = 0.937‡	r = 0.033<br>p = 0.647‡	r = -0.014<br>p = 0.842*
Diastolic blood pressure	r = -0.055<br>p = 0.443‡	r = -0.034<br>p = 0.635‡	r = -0.029<br>p = 0.688‡	r = -0.043<br>p = 0.551‡	r = 0.023<br>p = 0.744*
Mean arterial blood pressure	r = -0.031<br>p = 0.669‡	r = -0.014<br>p = 0.846‡	r = -0.015<br>p = 0.837‡	r = -0.011<br>p = 0.884‡	r = 0.008<br>p = 0.915*
Mean ocular perfusion pressure	r = -0.011<br>p = 0.988‡	r = 0.017<br>p = 0.811‡	r = 0.015<br>p = 0.836‡	r = 0.021<br>p = 0.773‡	r = 0.014<br>p = 0.844*
Heart rate	r = 0.049<br>p = 0.513‡	r = 0.041<br>p = 0.577‡	r = 0.030<br>p = 0.689‡	r = 0.065<br>p = 0.384‡	r = -0.050<br>p = 0.500*
Hemoglobin	r = -0.012<br>p = 0.865‡	r = 0.015<br>p = 0.841‡	r = 0.036<br>p = 0.618‡	r = -0.038<br>p = 0.603‡	r = 0.130<br>p = 0.073*
Hemoglobin A1c	r = 0.009<br>p = 0.902‡	r = 0.019<br>p = 0.786‡	r = 0.009<br>p = 0.902‡	r = 0.043<br>p = 0.550‡	r = 0.038<br>p = 0.593*
Body mass index	r = -0.054<br>p = 0.457‡	r = -0.058<br>p = 0.423‡	r = -0.044<br>p = 0.542‡	r = -0.084<br>p = 0.241‡	r = 0.023<br>p = 0.754*
Duration of diabetes	r = -0.002<br>p = 0.980‡	r = -0.045<br>p = 0.532‡	r = -0.034<br>p = 0.638‡	r = -0.066<br>p = 0.354‡	r = 0.009<br>p = 0.905*

Serum albumin	r = -0.083<br>p = 0.248‡	r = -0.088<br>p = 0.221‡	r = -0.058<br>p = 0.423‡	r = -0.151<br>p = 0.036‡	r = 0.132<br>p = 0.066*
eGFR	r = -0.125<br>p = 0.081‡	r = -0.115<br>p = 0.107‡	r = -0.104<br>p = 0.146‡	r = -0.130<br>p = 0.069‡	r = 0.014<br>p = 0.849*
Triglycerides	r = -0.027<br>p = 0.704‡	r = -0.027<br>p = 0.703‡	r = -0.026<br>p = 0.721‡	r = -0.028<br>p = 0.691‡	r = -0.004<br>p = 0.958*
Total cholesterol	r = 0.016<br>p = 0.826‡	r = 0.032<br>p = 0.660‡	r = 0.031<br>p = 0.670‡	r = 0.030<br>p = 0.685‡	r = 0.014<br>p = 0.850*
LDL-C/HDL-C	r = -0.003<br>p = 0.973‡	r = -0.006<br>p = 0.933‡	r = 0.000<br>p = 1.000‡	r = -0.020<br>p = 0.783‡	r = 0.027<br>p = 0.711*
Albumin index**	r = 0.166<br>p = 0.019‡	r = 0.195<br>p = 0.006‡	r = 0.164<br>p = 0.021‡	r = 0.249<br>p <0.001‡	r = -0.069<br>p = 0.330*

SFCT, subfoveal choroidal thickness; L/S ratio, ratio of luminal to stromal area; BCVA, best-corrected visual acuity; eGFR, estimated glomerular filtration rate; LDL-C/HDL-C, the ratio of low density lipoprotein cholesterol to high density lipoprotein cholesterol.

*Partial regression coefficients of correlation with the axial length as control variable.

†Partial regression coefficients of correlation with the age as control variable.

‡Partial regression coefficients of correlation with age and axial length as control variables.

**Albumin index = the ratio of urinal albumin to urinal creatinine

Supplementary Table S3. Differences in the choroidal structures between sexes.

	Men (n=126)	Women (n=74)	*P* value
Unadjusted SFCT, mean (SD), μm	288.9 (86.51)	286.6 (78.80)	0.847*
Adjusted SFCT, mean (SEM) [95%CI], μm	298.5 (6.51) [285.7-311.4]	270.2 (8.60) [253.2-287.2]	0.011†
Unadjusted Total choroidal area, mean (SD), $10^3 \mu m^2$	1656.7 (457.52)	1626.8 (418.63)	0.646*
Adjusted Total choroidal area, mean (SEM) [95%CI], $10^3 \mu m^2$	1704.5 (34.40) [1636.6-1772.3]	1545.4 (45.49) [1455.7-1635.1]	0.007†
Unadjusted Luminal area, mean (SD), $10^3 \mu m^2$	1102.2 (329.85)	107.7 (293.85)	0.582*
Adjusted Luminal area, mean (SEM) [95%CI], $10^3 \mu m^2$	1136.2 (24.98) [1086.9-1185.5]	1018.8 (33.03) [953.6-1083.9]	0.006†
Unadjusted Stromal area, mean (SD), $10^3 \mu m^2$	554.4 (138.82)	550.2 (136.38)	0.833*
adjusted Stromal area, mean (SEM) [95%CI], $10^3 \mu m^2$	568.3 (10.59) [547.4-589.2]	526.6 (13.99) [499.0-554.2]	0.021†
Unadjusted Luminal/Stromal ratio, mean (SD)	1.98 (0.269)	1.96 (0.248)	0.596*
Adjusted Luminal/Stromal ratio, mean (SEM) [95%CI]	1.99 (0.023) [1.95-2.04]	1.94 (0.03) [1.88-2.00]	0.145‡

SFCT, subfoveal choroidal thickness; SEM, standard error of the mean.
*unpaired-t test.
†General linear model where age and axial length were used as covariance. Covariates appearing in the model are evaluated at the following values: age=58.58, axial length=23.86.
‡ General linear model where axial length were used as covariance. Covariates appearing in the model are evaluated at the following values: axial length=23.86.

Supplementary Table S4: Correlation of choroidal structures with categorical variables*

	SFCT, μm	p	Total choroidal area, $10^3 \mu m^2$	p	Luminal area, $10^3 \mu m^2$	p	Stromal area, $10^3 \mu m^2$	p	L/S ratio	p
Type of diabetes, 1 (n=15)	270.4 (73.56)	0.396	1563.7 (387.33)	0.458	1029.6 (265.73)	0.423	534.2 (125.89)	0.585	1.93 (0.186)	0.481
2 (n=185)	289.5 (84.34)		1652.2 (447.16)		1097.9 (320.33)		554.4 (138.71)		1.98 (0.266)	
Hypertension present (n=138)	281.6 (77.09)	0.101	1599.3 (403.53)	0.027	1058.8 (286.32)	0.023	540.4 (127.52)	0.056	1.96 (0.251)	0.254
absent (n=62)	302.5 (95.48)		1748.8 (507.90)		1168.2 (366.44)		580.5 (155.28)		2.00 (0.281)	
Dyslipidemia present (n=132)	288.6 (83.25)	0.904	1651.6 (448.68)	0.791	1099.0 (327.48)	0.698	552.6 (133.33)	0.968	1.98 (0.283)	0.574
absent (n=68)	287.1 (84.73)		1634.0 (433.90)		1080.6 (295.99)		553.4 (146.54)		1.96 (0.212)	
Current smoking present (n=56)	288.5 (82.97)	0.961	1658.6 (444.76)	0.797	1100.2 (312.96)	0.836	558.4 (141.66)	0.725	1.97 (0.237)	0.924
absent (n=144)	287.9 (84.06)		1640.6 (443.35)		1089.8 (318.90)		550.7 (136.43)		1.97 (0.270)	
DME present (n=70)	305.6 (83.31)	0.029	1736.0 (450.22)	0.034	1148.1 (325.83)	0.070	587.9 (140.94)	0.008	1.95 (0.291)	0.436
absent (n=130)	278.6 (82.46)		1597.0 (432.54)		1063.0 (308.53)		534.0 (132.50)		1.98 (0.243)	
Type of DME† absent (n=130)	278.6 (82.46)	0.090	1597.0 (432.54)	0.072	1063.0 (308.53)	0.176	534.0 (132.50)	0.009	1.98 (0.243)	0.589
Sponge-like swelling (n=9)	282.4 (54.40)		1571.1 (283.34)		1051.8 (202.18)		519.3 (91.76)		2.04 (0.234)	
Cystoid macular edema (n=42)	302.0 (85.66)		1721.4 (458.61)		1139.4 (328.65)		582.0 (140.87)		1.95 (0.269)	
Serous retinal	324.4		1846.4		1212.8		633.6		1.91	

detachment (n=19)	(88.90)	(485.06)	(365.44)	(150.22)	(0.363)

SFCT = subfoveal choroidal thickness; L/S ratio = ratio of luminal to stromal area; DME = diabetic macular edema.
*Data were presented as mean (SD). Statistical analyses were performed with unpaired-t test except for the difference among type of DME which was done with one-way analysis of variance.
†Eyes with concurrent serous retinal detachment and other type of macular edema were categorized into serous retinal detachment group, and eyes with sponge-like swelling and cystoid macular edema were categorized into cystoid macular edema group.

Supplementary Table S5: Linear Regression Analysis with Choroidal Parameters as Dependent Variables and Diabetic Medications as Independent Variables*

	SFCT†, B (β) [95%CI] , μm	p	Total choroidal area‡, (β) [95%CI] , $10^3μm^2$	p	Luminal area**, β (95%CI) , $10^3μm^2$	p	Stromal area††, β (95%CI) , $10^3μm^2$	p	L/S ratio‡‡, β (95%CI)	p
insulins with medication	-9.7 (-0.057) [-30.8 to 11.5]	0.369	-56.1 (-0.063) [-167.6 to 55.3]	0.322	-51.7 (-0.081) [-133.7 to 30.3]	0.215	-9.1 (-0.033) [-42.6 to 24.5]	0.595	-0.053 (-0.100) [-0.127 to 0.022]	0.167
dipeptidyl peptidase-4 inhibitors with medication	7.7 (0.046) [-12.4 to 27.8]	0.450	39.3 (0.044) [-65.9 to 144.5]	0.462	14.3 (0.023) [-63.1 to 91.7]	0.364	26.0 (0.094) [-5.9 to 57.8]	0.110	-0.031 (-0.079) [-0.104 to 0.042]	0.404
glucagon-like peptide-1 analogues with medication	-14.5 (-0.032) [-68.8 to 39.5]	0.596	-34.7 (-0.014) [-317.6 to 248.2]	0.809	-45.0 (-0.026) [-253.1 to 163.1]	0.670	-3.2 (-0.004) [-88.0 to 81.6]	0.941	-0.112 (0.079) [-0.312 to 0.087]	0.268
biguanides with medication	8.9 (0.052) [-11.2 to 29.0]	0.383	36.8 (0.040) [-72.0 to 145.6]	0.506	25.9 (0.040) [-54.2 to 105.9]	0.524	15.9 (0.056) [-16.8 to 48.6]	0.338	0.028 (0.052) [-0.046 to 0.102]	0.460
sulfonylureas with medication	10.7 (0.054) [-14.5 to 35.9	0.404	48.1 (0.046) [-85.4 to 181.5]	0.478	26.4 (0.035) [-71.8 to 124.5]	0.597	16.5 (0.051) [-23.7 to 56.7]	0.419	0.000 (0.000) [-0.092 to	0.999

									0.091]	
thiazolidinediones with medication	10.9 (0.032) [-28.9 to 50.7]	0.589	53.5 (0.030) [-157.8 to 264.7]	0.618	39.6 (0.031) [-115.8 to 195.0]	0.616	18.7 (0.034) [-45.0 to 82.4]	0.563	-0.009 (-0.009) [-0.155 to 0.137]	0.899
alfa-glucosidase inhibitors with medication	13.1 (0.046) [-21.7 to 47.9]	0.459	126.9 (0.084) [-55.1 to 308.9]	0.171	108.6 (0.101) [-25.3 to 242.5]	0.111	17.9 (0.038) [-37.3 to 73.1]	0.523	0.083 (0.093) [-0.044 to 0.210]	0.200
sodium-glucose co-transporter 2 inhibitors with medication	4.1 (0.016) [-27.3 to 35.6]	0.383	17.9 (0.013) [-146.8 to 182.6]	0.831	25.2 (0.025) [-95.9 to 146.4]	0.682	-4.9 (-0.011) [-54.5 to 44.7]	0.845	0.097 (0.116) [-0.016 to 0.209]	0.091
Glinides with medication	-17.5 (-0.054) [-56.9 to 22.0]	0.383	-78.3 (-0.045) [-285.0 to 128.3]	0.455	-70.3 (-0.057) [-222.4 to 81.7]	0.362	-4.4 (-0.008) [-66.4 to 57.5]	0.888	-0.107 (-0.105) [-0.251 to 0.038]	0.146

SFCT = subfoveal choroidal thickness; L/S ratio = ratio of luminal to stromal area; DME = diabetic macular edema.
*Data were presented as B, unstandardized coefficients (β, standardized coefficients) [95% CI for B].
†Model was adjusted for age, axial length, sex, and stage of DR.
‡Model was adjusted for age, axial length, sex, stage of DR, presence of HT, and albumin index.
**Model was adjusted for age, axial length, sex, stage of DR, and presence of HT.
††Model was adjusted for age, axial length, stage of DR, and albumin index.
‡‡Model was adjusted for axial length and BCVA.

Supplementary Table S6: Subgroup analysis according to the systemic medical conditions*

	SFCT†, B (β) [95%CI] , μm	p	Total choroidal area‡, (β) [95%CI] , $10^3μm^2$	p	Luminal area**, β (95%CI) , $10^3μm^2$	p	Stromal area††, β (95%CI) , $10^3μm^2$	p	L/S ratio‡‡, β (95%CI)	p
Calcium channel blockers***	-6.1 (-0.039) [-31.3 to 19.1]	0.634	-115.4 (-0.143) [-248.4 to 17.6	0.088	-49.2 (-0.086) [-143.1 to 44.7]	0.302	-23.4 (-0.091) [-64.3 to 17.6]	0.261	-0.102 (-0.133) [-0.187 to 0.017]	0.091
Angiotensin receptor blockers***	0.992 (0.006) [-24.7 to 26.7]	0.939	9.6 (-0.012) [-11.9 to 138.5]	0.884	15.3 (0.027) [-80.5 to 111.0]	0.302	-16.8 (-0.066) [-55.8 to 222.0]	0.396	0.062 (0.125) [-0.025 to 0.150]	0.160
Angiotensin converting enzyme inhibitors***	0.971 (0.004) [-42.8 to 44.6]	0.717	4.0 (0.003) [-215.5 to 223.5]	0.971	-28.8 (-0.028) [-191.8 to 134.1]	0.727	20.2 (0.045) [-47.5 to 87.3]	0.557	-0.126 (-0.143) [-0.277 to 0.024]	0.098
Beta-blockers***	-8.7 (-0.039) [-43.3 to 25.9]	0.621	-58.1 (-0.050) [-231.3 to 115.0]	0.508	-21.8 (-0.026) [-150.6 to 106.9]	0.738	-31.2 (-0.084) [-84.3 to 22.0]	0.249	0.094 (0.130) [-0.024 to 0.212]	0.118
Diuretic agents***	-3.2 (-0.016) [-35.5 to 29.1]	0.845	29.8 (0.028) [-131.8 to 191.5]	0.716	13.9 (0.018) [-106.3 to 134.1]	0.819	22.6 (0.067) [-27.0 to 72.2]	0.369	-0.023 (-0.035) [-0.135 to 0.089]	0.687

Statins†††	-10.5 (-0.063) [-34.4 to 13.4]	0.387	-80.8 (-0.090) [-209.4 to 47.9]	0.216	-47.7 (-0.073) [-144.4 to 49.0]	0.331	-23.1 (-0.087) [-59.9 to 13.7]	0.217	-0.025 (-0.044) [-0.115 to 0.066]	0.591

SFCT = subfoveal choroidal thickness; L/S ratio = ratio of luminal to stromal area.
*Data were presented as B, unstandardized coefficients (β, standardized coefficients) [95% CI for B].
†Model was adjusted for age, axial length, sex, and stage of DR.
‡Model was adjusted for age, axial length, sex, stage of DR, presence of HT, and albumin index.
**Model was adjusted for age, axial length, sex, stage of DR, and presence of HT.
††Model was adjusted for age, axial length, stage of DR, and albumin index.
‡‡Model was adjusted for axial length and BCVA.
***Analysis for participants with hypertension.
†††Analysis for participants with dyslipidemia.

Supplementary Figure S1

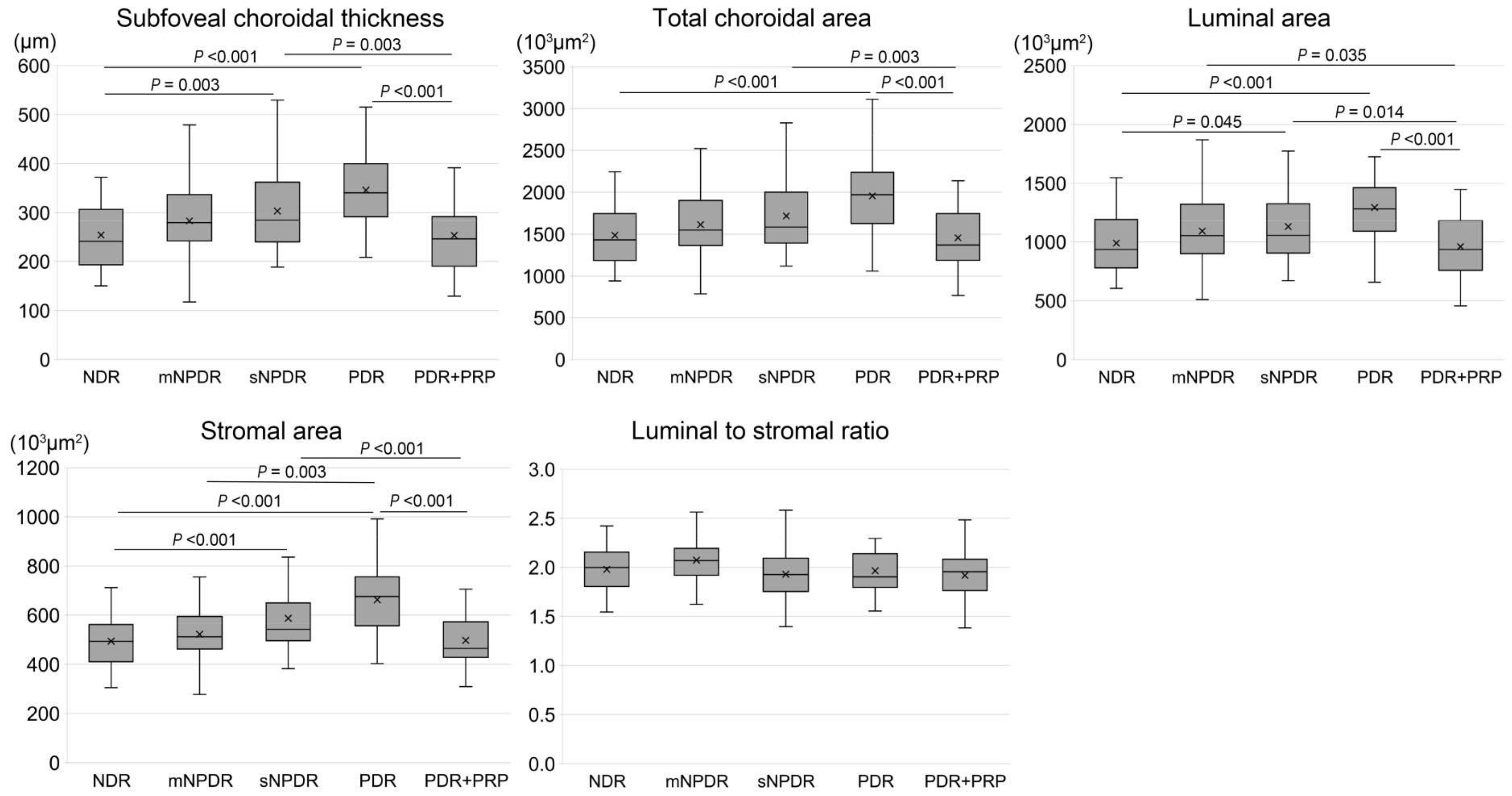

Fig. S1. Choroidal structural parameters for the eyes with different stages of diabetic retinopathy.
Subfoveal choroidal thickness, total choroidal area, luminal area, and stromal area were significantly larger at more advanced stage of DR, and smaller in eyes with PRP than those without. The luminal to stromal ratio is not significantly different among the different stages of DR. Boxes show the range of upper and lower quartiles. The horizontal line and the X in the boxes indicate the median and the mean, respectively. Whiskers extending from the box show the minimum and maximum values. *P* values represent the results of Bonferroni multiple comparisons test following a general linear model with the adjustments for age and axial length. NDR, no diabetic retinopathy; mNPDR, mild to moderate nonproliferative diabetic retinopathy; sNPDR, severe NPDR; PDR, proliferative diabetic retinopathy; PRP, panretinal photocoagulation.
